# Seedling Emergence and Phenotypic Response of Common Bean Germplasm to Different Temperatures under Controlled Conditions and in Open Field

**DOI:** 10.3389/fpls.2016.01087

**Published:** 2016-08-02

**Authors:** Antonio M. De Ron, Ana P. Rodiño, Marta Santalla, Ana M. González, María J. Lema, Isaura Martín, Jaime Kigel

**Affiliations:** ^1^Biology of Agrosystems, Misión Biológica de Galicia, National Spanish Research CouncilPontevedra, Spain; ^2^Sistemas Agroforestales, Unidad Asociada a la Misión Biológica de Galicia (CSIC)Pontevedra, Spain; ^3^Phytopathological Station do Areeiro, Provincial ChamberPontevedra, Spain; ^4^National Center for Plant Genetic Resources, National Institute for Agricultural and Food Research and TechnologyAlcalá de Henares, Spain; ^5^The Robert H. Smith Faculty of Agriculture, Food and Environment, Hebrew University of JerusalemRehovot, Israel

**Keywords:** low temperature tolerance, *Phaseolus vulgaris* L., plant breeding, plant genetic resources, seedling emergence, yield

## Abstract

Rapid and uniform seed germination and seedling emergence under diverse environmental conditions is a desirable characteristic for crops. Common bean genotypes (*Phaseolus vulgaris* L.) differ in their low temperature tolerance regarding growth and yield. Cultivars tolerant to low temperature during the germination and emergence stages and carriers of the grain quality standards demanded by consumers are needed for the success of the bean crop. The objectives of this study were (i) to screen the seedling emergence and the phenotypic response of bean germplasm under a range of temperatures in controlled chamber and field conditions to display stress-tolerant genotypes with good agronomic performances and yield potential, and (ii) to compare the emergence of bean seedlings under controlled environment and in open field conditions to assess the efficiency of genebanks standard germination tests for predicting the performance of the seeds in the field. Three trials were conducted with 28 dry bean genotypes in open field and in growth chamber under low, moderate, and warm temperature. Morpho-agronomic data were used to evaluate the phenotypic performance of the different genotypes. Cool temperatures resulted in a reduction of the rate of emergence in the bean genotypes, however, emergence and early growth of bean could be under different genetic control and these processes need further research to be suitably modeled. Nine groups arose from the Principal Component Analysis (PCA) representing variation in emergence time and proportion of emergence in the controlled chamber and in the open field indicating a trend to lower emergence in large and extra-large seeded genotypes. Screening of seedling emergence and phenotypic response of the bean germplasm under a range of temperatures in controlled growth chambers and under field conditions showed several genotypes, as landraces 272, 501, 593, and the cultivar Borlotto, with stress-tolerance at emergence, and high yield potential that could be valuable genetic material for breeding programs. Additionally, the potential genetic erosion in genebanks was assessed. Regarding bean commercial traits, under low temperature at sowing time seed reached larger size, and crop yield was higher compared to warmer temperatures at the sowing time. Therefore, early sowing of bean is strongly recommended.

## Introduction

The common bean (*Phaseolus vulgaris* L.) is native to the Americas where two major domestication centers and gene pools have been described, Andean and Mesoamerican, which differ in their adaptation to different climatic and eco-geographic conditions. Differences in the seed type and size are clear between both genetic pools (Singh et al., 1991; Santalla et al., 2001), having the Andean varieties larger seeds than the Mesoamerican ones. The diffusion of the common bean out of its American domestication centers appears to have been very complex, involving numerous introductions into different continents along a range of agrosystems. Several geographic regions have been proposed as secondary centers of diversification, such as Europe (Santalla et al., 2002; Angioi et al., 2010; Gioia et al., 2013), central-eastern and southern Africa, Brazil, and China (Bellucci et al., 2014). However, once out of the Americas, the spatial isolation between the Mesoamerican and Andean gene pools was not maintained, thus providing increased potential for their hybridization, and introgression. In Europe, this issue is highly relevant for breeding programs. Indeed, their hybridization has led to the recombination of the Mesoamerican and Andean traits resulting in novel and useful genotypes and phenotypes adapted to contrasting environmental conditions (i.e., resistance to biotic and abiotic stress; Rodiño et al., 2006; Angioi et al., 2010; Blair et al., 2010; Santalla et al., 2010). In contrast, various studies suggest that in other regions the introgression between these gene pools appears to be less relevant than in Europe (De Ron et al., 2015).

Early breeding efforts primarily focused on improved disease resistance and adaptation to local environments, with later efforts focused on improved seed quality, improved plant architecture, and breeding for yield (Duc et al., 2015). Yield *per-se*, tolerance to drought, adaptation to poor soils, and nutritional quality are priorities of bean breeders since the 1990s (De Ron et al., 2015). Seed germination and seedling emergence in the small seeded Mesoamerican genotypes is generally faster than that in the Andean ones, and this phenotypic trait has been used to distinguish between the two genetic pools (White and Montes, 1993). Faster emergence may reflect both genetic variation for adaptation to specific environments and effects of seed size in emergence. Seed size has been recognized as a factor affecting bean germination (Hanley et al., 2003; Kaya et al., 2008) and is probably related to water uptake, a key process in seedling emergence (Bewley, 1997). High seed vigor, good germination, and emergence are prerequisites for successful direct sowing in common bean. Thus, a better understanding of the genetics of the processes regulating germination and early growth under different conditions is important not only as a contribution to the knowledge of this species, but also has direct applications in plant breeding, and for germplasm conservation and regeneration.

The bean crop experienced a quick adaptive radiation throughout Europe in the Sixteenth Century (Zeven, 1997), where it was distributed through very different edapho-climatic environments. The microclimate of the cultivated areas, located at different latitudes, and altitudes, could have a strong influence on the recent evolution of this crop (Escribano et al., 1994; Santalla et al., 2002, 2010; Casquero et al., 2006; González et al., 2006; Papa et al., 2006; Rodiño et al., 2006). Several studies showed that a number of varieties with relevance for niche markets still survive on-farm in marginal areas of European countries (Zeven et al., 1999; Eichenberger et al., 2000; Rodiño et al., 2001, 2003, 2009; Negri and Tosti, 2002; Sicard et al., 2005) and in their areas or origin (De Ron et al., 2004; Galván et al., 2006). Common bean is adapted to relatively humid and cool climatic conditions with optimal average daily temperature for reproductive development ranging from 20 to 25°C (Wantanbe, 1953). Temperatures >30°C during the day or >20°C at night result in yield reduction (Hardwick and Andrews, 1980; Rainey and Griffiths, 2005), and seeds germinate poorly below 15°C (Kotowski, 1926; Kigel et al., 2015). Thus, it is necessary to restrict field sowing of beans to warm climates or to delay sowing until the soil is warm enough for satisfactory emergence in cool climates (Hardwick, 1972). Moreover, beans that are slow to germinate are also likely to be slow in growth (Kooistra, 1971). The physiological response of common bean to high temperature stress has been primarily studied through the use of controlled environmental testing in greenhouses and growth chambers (Porch, 2006). However, the long-term goal of breeding for stress tolerance is the development of germplasm with improved field adaptation to different temperatures. Therefore, in order to make maximum use of the available growing period, genotypes must be developed that are tolerant to low temperature during germination and early growth.

Seed germination is the process that commences with uptake of water by the dry seed—i.e., imbibition, and terminates with emergence of the seedling. Thus, the process involves two temporal stages, namely the germination stage and the emergence, or seedling-growth stage (Bewley and Black, 1985; Bewley, 1997). The emergence of the radicle marks the end of the first stage and the onset of the second. The sooner the radicle protrudes through the seed coat, the faster is the emergence. Fast seed germination is considered an important adaptive trait marking a quick transition to the growth phase in the life-cycle of a plant. The time taken for the germination process to be completed is one of the important parameters of seed quality (Copeland and McDonald, 1995; Dutt and Geneve, 2007). Vigorous, rapid, as well as uniform germination and emergence under diverse environmental conditions is a desirable attribute for seedling growth and, ultimately, grain yield in food legumes and cereal crops such as bean, rice, wheat, maize. Crop species vary widely in how fast their seeds germinate, the rate of emergence being the result of the interaction between the seed genotype and specific environmental, or ecological factors (Hernández-Nistal et al., 1989; Alonso-Blanco et al., 2003; Schmuths et al., 2006).

In past years, substantial progress was achieved by plant breeders in adapting crops such as maize, tomato, soybean, and common bean to suboptimal temperatures (Dickson, 1971; Holmberg, 1973; Skrdla and Mock, 1978; Patterson and Payne, 1983). Ideally, the best cold-tolerant genotypes should have successful water imbibition, germination, and emergence at low temperature (Kemp, 1978; Garcia-Huidobro et al., 1982; Dickson and Boettger, 1984a,b; Gummerson, 1986). The interval from seedling to maximum growth and blooming of bean plants should be shortened by selecting lines capable of rapid early growth during periods of low temperature following sowing.

The cultivation of dry bean in South Europe has traditionally taken place with sowings form April–June. But summer cultivation increases production costs by demanding greater irrigation and more tillage because of weed proliferation. Moreover, summer cultivation increases the likelihood of harvest coinciding with the onset of the rainy season, leading to crop failure, and yield losses. Cultivation in the spring period, however, is restricted because low temperatures at sowing delay germination, seedling emergence, and early growth. The alternative, therefore, is to use genotypes tolerant to low temperature at the germination, emergence, and early growth stages. Yet, little research has been done to breed this type of dry bean genotypes. Thus, further identification of cold tolerant genotypes already reported in a few instances (Dickson, 1971; Kooistra, 1971; Bannerot, 1979; Hardwick and Andrews, 1980; Dickson and Boettger, 1984a,b; Scully and Waines, 1987) is necessary.

The purpose of germination testing in genebanks and breeder collections is to provide information on the comparative and foreseeable field planting value of different seed samples. In the case that field testing seed emergence, which can be affected by the field conditions (Ellis et al., 1985), failed more than expected according the tests of germination, there may be a loss of genetic material of gene bank accessions that will imply a process of genetic erosion.

The objectives of this study were (i) to screen the seedling emergence and the phenotypic response of bean germplasm under a range of temperatures in controlled chamber and field conditions to display stress-tolerant genotypes with good agronomic performances and yield potential, and (ii) to compare the emergence of bean seedlings under controlled environment and in open field conditions to assess the efficiency of genebanks standard germination tests for predicting the performance of the seeds in the field.

## Materials and methods

### Plant material

Twenty-eight accessions were used in this study—21 landraces, five breeding lines, and two cultivars. The seeds were maintained in the Misión Biológica de Galicia-Spanish National Research Council (MBG–CSIC, Pontevedra, Spain) germplasm facilities at 4°C and 40% RH (relative humidity; Table 1).

**Table 1:** **Origin, seed size, genetic pool, market class, and seed age of the common bean genotypes studied**.

**Genotype^a^**	**Geographical origin**	**Seed size (g 100 seed^−1^)**	**Genetic pool**	**Market class^b^**	**Seed age (years)**
**LANDRACES**					
200	43° 07′ N, 8° 55′ W, 247 masl	57	Andean	White kidney	1
272	42° 17′ N, 8° 12′ W, 380 masl	36	Andean	Purple caparron	4
391	42° 13′ N, 8° 16′ W, 545 masl	61	Andean	Red pinto	2
399	43° 29′ N, 6° 05′ W, 37 masl	70	Mesoamerican	Large great northern	2
413	43° 26′ N, 6° 06′ W, 420 masl	79	Andean	Favada pinto	4
419	42° 34′ N, 8° 53′ W, 6 masl	73	Mesoamerican	Large great northern	1
452	43° 31′ N, 7° 06′ W, 298 masl	79	Andean	Favada	1
489	42° 39′ N, 2° 32′ W, 723 masl	40	Andean	Guernikesa	4
501	43° 14′ N, 2° 08′ W, 312 masl	43	Andean	Negro brillante	2
573	40° 30′ N, 5° 45′ W, 959 masl	35	Mesoamerican	Common pinto	1
587	41° 13′ N, 5° 29′ W, 810 masl	37	Mesoamerican	Great northern	1
593	41° 23′ N, 2° 10′ E, 8 masl	30	Mesoamerican	Hook	1
623	41° 23′ N, 2° 10′ E, 8 masl	33	Mesoamerican	Hook	1
837	43° 11′ N, 8° 44′ W, 135 masl	57	Andean	Canario bola	9
838	43° 15′ N, 8° 54′ W, 50 masl	66	Andean	White kidney	1
839	43° 15′ N, 8° 53′ W, 60 masl	81	Andean	Favada	1
842	43° 15′ N, 8° 53′ W, 60 masl	66	Andean	White kidney	9
917	43° 41′ N, 7° 30′ W, 40 masl	87	Andean	Favada	1
921	43° 41′ N, 7° 30′ W, 40 masl	77	Andean	Favada	1
924	43° 41′ N, 7° 30′ W, 40 masl	75	Andean	Favada	1
1058	41° 20′ N, 7° 45′ W, 500 masl	47	Mesoamerican	Great northern	1
**BREEDING LINES**^c^					
Andecha	43° 29′ N, 5° 26′ W, 16 masl	76	Andean	Favada	4
Bonafema	43° 29′ N, 5° 26′ W, 16 masl	76	Andean	Favada	1
Collacia	43° 29′ N, 5° 26′ W, 16 masl	68	Andean	Favada	1
Montcau	41°23′ N, 2° 07′ E, 63 masl	32	Mesoamerican	Hook	1
Peregrina	43° 23′ N, 8° 08′ W, 178 masl	46	Mesoamerican	Great northern	1
**CULTIVARS**					
Borlotto	Vilmorin (France)	54	Andean	Cranberry	1
Fukuryu	Agricultural Station (Japan)	56	Andean	Cranberry	1

a *All the genotyes are from Spain, with the exception of the last two ones*.

b *Santalla et al. (2001)*.

c *Originated from traditional landraces by individual selection*.

### Controlled climatic chamber experiments

The experiments were carried out at the MBG-CSIC using a climatic chamber set at different day-lengths and temperatures that represent conditions of North-western Spain: tc_1_: 14/10 h (day/night) at 14/10°C and 60/80% RH representing field conditions in April (weighted average: 11.5°C, 68.3% RH); tc_2_: 15/9 h at 17/12°C and 60/80% RH representing conditions in May (weighted average: 15.1°C, 67.5% RH); and tc_3_: 16/8 h at 22/15°C and 60/80% RH representing conditions in June (weighted average: 19.7°C, 66.7% RH). Light was provided by seven very high output (VHO) fluorescent lamps with a photosynthetic photon flux (PPF) of 228 μmol m^−2^ s^−1^.

A randomized complete block design with three replications was used. Ten seeds of each genotype were sown in sterile peat in plastic containers (30 × 20 × 12 cm), with a plant to plant (seed to seed) distance of 2.5 cm, and row to row distance of 5 cm. Monitoring of emergence was carried out during ca. 30 days, starting 4 days after the beginning of the experiments. Seedlings with a hypocotyl-radicle axis >3 cm were considered as emerged. Proportion of emergence (% of sown seeds) and time to emergence (days from sowing to seedling emergence of all seeds sown) were measured.

### Field experiments

The open field experiments were conducted in the experimental farm of the MBG-CSIC at Pontevedra, Spain (42° 24′ N latitude, 8° 38′ W longitude, 40 masl 14°C average annual temperature, and 1600 mm annual rainfall). The soil, developed from granitic rocks, has a sandy loam texture, and a granular structure. It is classified as Humic Cambisol according to FAO criteria (FAO-ISRIC-ISSS, 1998).

To test temperature effects in the field, seeds were sown by hand at: (a) low temperature conditions (tf_1_) on 10 April 2007; (b) moderate temperature conditions (tf_2_) on 4 May 2007; and c) warm temperature conditions (tf_3_) on 7 June 2007 (Table S1). Field trials were arranged according to a completely randomized block design with 30 plants per sowing treatment and two replications (crop density of 50000 plant ha^−1^). Single row plots were 3.8 m in length and 0.8 m spaced. Plants were watered as needed, using drip irrigation. The following traits were measured: Emergence time (days) and emergence proportion (%); expansion of the first trifoliate leaf (days); plant height at 10 and 20 days from emergence (centimeters); early plant vigor (from 1 to 9 scale); days from sowing to first open flower, beginning of flowering (50% of plants with at least an open flower), end of flowering (days), and physiological mature pod (days); dry seed weight (g 100 seed^−1^) and seed dimensions (millimeters). Seeds per pod and pods per plant were determined on a plot average basis. Five plants were selected from the center of the plots for the estimation of grain yield (expressed in kg ha^−1^).

### Soil properties

Five soil samples were collected before sowing in the field. Samples were air dried and passed through a 2 mm sieve prior to analysis. Soil organic matter was estimated by weight loss after ignition (Schulte and Hopkins, 1996). Soil pH in H_2_O and 0.1 M KCl were measured (solution ratio 1:2.5). Phosphorus availability was measured by a modification of the Olsen method (Olsen and Dean, 1965). Exchangeable cations were extracted with 1 M NH_4_Cl (Peech et al., 1947) and determined by atomic absorption spectrophotometry. Effective cation exchange capacity (ECEC) was calculated as the sum of base cations plus aluminum. The soil had moderate levels of total organic matter (4.2%) due to the long history of cultivation in the experimental plot and had an acidic reaction (pH = 5.7) and significant levels of exchangeable aluminum, as expected in soil derived from granitic like rock. ECEC was relatively low, although the potassium levels were medium to high. High phosphorus availability was due to fertilization with organic manure for many years in the past. Overall, soil analyses showed that the experimental plot was representative of most agricultural soils intensively cultivated in the NW of Spain. The Table S2 summarizes the chemical properties of the soil of the experimental plot.

### Data analyses

Maximum, minimum, average temperatures and RH were daily measured in the controlled chamber and in the open field trials. The statistical analyses were conducted using the general linear model (GLM) procedure of the SAS 2000 statistical package. The least significant difference (LSD) (*P* ≤ 0.05) was used to evaluate differences among genotype means. Standard errors and coefficients of variation were also computed (Steel et al., 1997).

Principal component analysis (PCA) was performed by NTSYS-pc v.2.10 (Rohlf, 2000) and the free software R^mo^ modified by García-Pérez (2005) to display the ordination of the genotypes under both environments. The variables used in the PCA were emergence proportion and time to emergence at the three temperature levels (t1, t2, t3) in the controlled chamber and in the open field. According to the scree diagram most of the variation was explained by the first (PC1), second (PC2), and third (PC3) principal components. PC1 and PC2 accounted for 56% of variation and were used for plotting the 28 genotypes.

## Results

### Screening in controlled chamber

Marked differences in emergence proportion and time to emergence were found among genotypes at the different temperatures in the controlled chamber and in the open field sowings, respectively (Table 2). In the controlled chamber emergence proportion ranged from 30.0 to 96.7%, with an average of 72.9% at low temperature (tc1 = 14/8°C). Under 17/12°C (tc2) and 22/15°C (tc3), some genotypes reached 100% emergence, the averages were 88.2 and 94.4%, respectively. Seedling emergence was delayed under the lower temperature in controlled conditions averaging 27.2 d at 14/8°C (tc1), but emergence time was drastically reduced to 7.8 and 4.6 d when temperature was increased to 17/12°C (tc2) and 22/15°C (tc3), respectively. Effects of genotype, temperature, and genotype × temperature interaction on emergence proportion and time to emergence were significant (Table 3), indicating differential and non-uniform responses of genotypes to temperature at the emergence stage.

**Table 2:** **Emergence proportion and time to emergence of the common bean genotypes studied, grown under three different temperatures in controlled chamber, and open field**.

**Genotypes**	**Emergence proportion (%)**						**Emergence time (d)**					
	**Controlled chamber**			**Open field**			**Controlled chamber**			**Open field**		
	**tc1**	**tc2**	**tc3**	**tf1**	**tf2**	**tf3**	**tc1**	**tc2**	**tc3**	**tf1**	**tf2**	**tf3**
200	86.7	100.0	96.7	50.0	50.0	50.0	28.3	7.3	4.7	11.5	9.5	12.0
272	96.7	86.7	96.7	58.3	58.3	50.0	23.0	8.0	3.7	15.5	9.5	12.0
391	53.3	90.0	83.3	50.0	50.0	50.0	29.7	8.0	4.0	10.0	9.0	13.0
399	63.3	73.3	86.7	75.0	50.0	50.0	30.3	10.0	9.3	13.5	8.5	12.5
413	96.7	100.0	100.0	50.0	50.0	50.0	27.0	7.3	4.7	11.5	8.5	12.5
419	73.3	90.0	90.0	75.0	50.0	40.0	29.0	8.0	4.7	13.5	8.0	13.0
452	80.0	93.3	96.7	50.0	50.0	20.0	26.3	7.3	4.3	15.0	9.0	14.0
489	70.0	70.0	100.0	50.0	50.0	41.7	26.3	7.7	4.0	13.0	10.0	13.0
501	93.3	96.7	100.0	50.0	50.0	35.0	23.7	7.3	4.7	17.0	9.0	14.0
573	33.3	80.0	83.3	50.0	45.0	43.3	29.3	8.0	3.7	12.5	13.5	13.5
587	63.3	100.0	96.7	50.0	50.0	31.7	26.0	8.3	3.7	16.5	9.5	11.0
593	90.0	96.7	100.0	50.0	40.0	50.0	24.3	7.3	4.0	14.5	14.0	13.0
623	30.0	93.3	100.0	35.0	35.0	48.3	27.0	7.3	4.3	16.5	19.0	14.0
837	63.3	80.0	96.7	50.0	50.0	50.0	30.7	8.0	5.0	11.5	8.5	12.5
838	50.0	93.3	86.7	38.3	40.0	38.3	31.3	8.3	5.0	16.0	14.0	13.5
839	33.3	90.0	76.7	31.7	56.7	50.0	30.3	7.3	4.3	17.5	14.5	13.0
842	83.3	90.0	100.0	40.0	50.0	50.0	27.7	7.3	4.3	17.5	10.0	13.0
917	63.3	80.0	86.7	40.0	50.0	20.0	24.7	7.3	4.7	18.0	8.0	14.0
921	70.0	76.7	93.3	31.7	20.0	11.7	26.7	8.0	4.7	19.5	17.5	14.0
924	76.7	76.7	93.3	36.7	21.7	21.7	27.0	7.3	4.3	19.0	23.5	14.0
1058	86.7	90.0	100.0	58.3	58.3	50.0	26.0	7.3	4.7	12.5	10.0	12.5
Andecha	96.7	96.7	100.0	45.0	50.0	31.7	27.3	7.3	4.7	16.0	8.5	13.5
Bonafema	86.7	86.7	96.7	23.3	35.0	35.0	26.7	7.3	5.0	20.5	12.5	13.0
Collacia	53.3	66.7	90.0	16.7	36.7	38.3	29.3	11.0	4.3	20.0	14.0	13.5
Montcau	96.7	100.0	96.7	50.0	58.3	50.0	24.0	7.3	4.0	13.5	8.0	6.0
Peregrina	83.3	93.3	96.7	23.3	33.3	26.7	26.7	8.3	4.0	20.0	13.0	14.0
Borlotto	93.3	100.0	100.0	50.0	50.0	50.0	28.7	7.0	4.5	10.5	8.0	12.0
Fukuryu	73.3	80.0	100.0	33.3	50.0	50.0	25.0	7.3	4.7	17.5	9.0	13.0
Average	72.9	88.2	94.4	45.1	46.0	40.5	27.2	7.8	4.6	15.4	11.3	12.8

**Table 3:** **Mean squares and coefficient of variation (CV) from the analysis of variance for the emergence proportion and time to emergence of the common bean genotypes studied, grown under three different temperatures in controlled chamber**.

**Source of variation**	**Df^a^**	**Emergence proportion (%)**	**Emergence time (d)**
Replications	2	64.0	4.9
Genotype	27	897.0^**^	10.7^**^
Temperature	2	10278.4^**^	12386.3^**^
Genotype x temperature	54	350.7^**^	4.8^*^
Error	110	147.9	3.2
CV (%)		14.3	13.4

a *Df, Degrees of freedom*.

*, ** *significant at P ≤ 0.05, P ≤ 0.01, respectively*.

### Screening in open field trials

In terms of average daily temperatures, the high temperature trials (12.7–21.3°C), and the low temperature trials (9.3–20.4°C) overlap. However, the low and high temperature trials were still on the highest end of ideal common bean growing maximum and minimum temperatures (20–25 and 15°C) and thus probably experienced some temperature stress.

Analysis of variance of the 28 genotypes in the three open field experiments with different temperature conditions at sowing time is shown in Table 4. All the traits displayed significant differences among genotypes and among sowing times characterized by different temperatures, while significant genotype × sowing time interaction was only observed for dry seed weight, and length, pods per plant and yield. Only six genotypes showed emergence proportion higher than 50% in the earlier sowings at lower temperatures (tf1 and/or tf2), none under the warmer temperatures at the later sowing (tf3), averaging 45.1, 46.0, and 40.5%, respectively, at these sowing dates (Table 4). Differences in emergence time were smaller compared to the controlled chamber experiment. Emergence time was reduced from 15.4 d in the earlier, and colder conditions to 11.3 and 12.8 d in the later warmer conditions (*P* = 0.05). Early sowing in April associated with lower temperatures (tf1) significantly delayed by 4–5 d seedling emergence, first trifoliate leaf expansion, and beginning of flowering and by about 14–16 d the end of flowering and physiological maturity, compared to sowing in May (tf2). Sowing at warmer temperatures in June further reduce seed weight and plant yield. These results indicate that early sowing under lower temperatures produced taller plants with larger seeds and higher yield than later sowings under higher temperatures. In fact, the length of the vegetative and reproductive cycle stages was progressively reduced from the colder (tf1) to the warmer (tf2 and tf3) conditions. Thus, under lower temperatures plants had more time for growth, as well as for pod and seed set and maturation, resulting in larger seeds, and higher plant yield. The Table 5 displays the crop yield of the common bean genotypes studied, grown under three different temperatures in open field. The yield was clearly higher when sowing and growing under tf1 and tf2 than tf3 for all the genotypes, averaging 1188, 849, and 419 kg ha^−1^, respectively. Best performer was the landrace 419 (3118 kg ha^−1^ at tf1) and the worst was the Japanese cultivar Fukuryu (149 kg ha-1 at tf1), the latter probably due to lack of adaptation to the growing area. Interestingly, the 10 top yielding genotypes corresponded to tf1, while the 10 worst genotypes were sown and grown at tf3, except the aforementioned cultivar Fukuryu (tf1). The performance of some genotypes was consistent through the different temperature conditions, such as 399, 501, 587, and Borlotto.

**Table 4:** **Mean squares, coefficient of variation (CV) and genotype means from the analysis of variance of agronomic traits of the common bean genotypes studied, grown under three different conditions in open field**.

**Trait**	**Sources of variation**						**Mean**			
	**Replications**	**Genotype (G)**	**Temperature (T)**	**G × T**	**Error**	**CV (%)**	**tf1**	**tf2**	**tf3**	**LSD^b^**
Emergence time (d)	20.02	31.02^**^	236.74^**^	11.14	7.50	20.81	15.36a	11.28c	12.82b	1.26
Emergence proportion (%)	26.46	541.97^**^	490.51^*^	146.86	151.19	28.04	45.06ab	46.01a	40.48b	4.59
Leaf expansion (d)	3.72	15.55^*^	327.25^**^	10.14	7.88	14.32	22.36a	17.84b	18.61b	1.21
Plant height-10 days (mm)	5.28	58.86^**^	3333.78^**^	31.76	25.35	19.89	19.12c	33.95a	22.85b	2.13
Plant height-20 days (mm)	7.86	61.14^**^	5004.26^**^	18.87	29.61	12.85	53.30a	37.68b	35.70c	1.66
Early vigor (1-9)	0.53	6.43^**^	60.06^**^	2.22	2.54	28.52	6.46a	5.87b	4.42c	0.57
First open flower (d)	25.14	378.28^**^	1139.59^**^	22.74	24.69	8.66	61.96a	57.21b	52.95c	1.80
Beginning of flowering (d)	18.34	458.27^**^	1267.97^**^	33.01	23.89	7.87	66.79a	62.18b	57.23c	2.18
End of flowering (d)	23.54	77.34^**^	18036.40^**^	12.63	9.02	2.77	124.98a	111.14b	88.26c	1.35
Pod physiological maturity (d)	7.04	90.77^**^	16857.12^**^	13.36	12.74	3.15	130.20a	114.02b	94.72c	1.39
Dry seed weight (g 100 seed^−1^)	78.38	1863.09^**^	1825.58^**^	89.76^**^	43.11	11.15	63.75a	60.32a	51.99b	3.66
Seed length (mm)	4.74^*^	65.45^**^	12.98^**^	1.78^**^	0.72	5.14	16.96a	16.62a	16.09b	0.51
Seed width (mm)	0.14	3.69^**^	3.97^**^	0.12	0.07	3.31	8.54a	8.33b	7.99c	0.13
Seed thickness (mm)	0.02	4.74^**^	2.40^**^	0.10	0.13	5.91	6.28a	6.15b	5.80c	0.12
Seeds pod^−1^	1.92	4.94^**^	4.13^**^	0.61	0.55	17.64	4.35a	4.31a	3.89b	0.30
Pods plant^−1^	119.76	2108.50^**^	10732.38^**^	804.45^**^	321.42	55.00	31.23b	45.74a	20.14c	0.32
Yield (kg ha^−1^)	67054.50	4086.89^**^	55260.74^**^	3145.16^**^	1841.85	0.53	1188.20b	849.10a	419.00c	2.28
Df^a^	1	27	2	54	56					

*, ** *significant at P ≤ 0.05, P ≤ 0.01, respectively*.

a *Df, degrees of freedom*.

b *Least Significant Difference. Means follow for the same letter are not significant different at P ≤ 0.05*.

**Table 5:** **Crop yield of the common bean genotypes studied, grown under three different temperatures (tf1, tf2, and tf3) in open field**.

**Genotype**	**Yield (kg ha^−1^)**		
	**tf1**	**tf2**	**tf3**
200	1025	946	373
272	2236	811	448
391	1463	955	404
399	1594	1152	474
413	468	477	465
419	3118	1092	465
452	818	1128	435
489	367	431	nm^*^
501	2738	1395	425
573	777	907	355
587	1643	1168	482
593	1072	962	502
623	825	734	382
837	1084	862	393
838	478	544	305
839	1551	1567	445
842	420	602	349
917	1599	1234	356
921	1199	443	339
924	1055	706	526
1058	2861	1006	432
Andecha	1533	785	343
Bonafema	370	461	379
Collacia	884	921	319
Montcau	626	496	428
Peregrina	851	491	350
Borlotto	471	1157	717
Fukuryu	149	325	429
Average	1188	849	419

* *nm, not measured*.

### Comparison of temperature effect on chamber and field trials

The analysis of the emergence process was assessed in controlled chamber and in open field under three different temperature ranges by two variables: Time to emergence and proportion of emergence. Maximum, minimum, and the average values of these variables in chamber and field are shown in Table S3. Decrease in number of days to emergence and increase in emergence proportion when temperature increased occurred in the chamber trials but were less clear in the field (Table 4). Under favorable conditions (tc2 and/or tc3) 13 genotypes reached 100% emergence in the growth chamber. In the open field, in contrast, only four genotypes showed emergence higher than 50% at tf2 and none at tf3, while emergence level was similar at tf1 and tf2, and higher than at tf3. Emergence time across genotypes was relatively stable at the lower temperatures (tc1) in the growth chamber, ranging from 23.0 to 31.3 d, but in the field the range was 10.0 to 20.5 d in the first sowing (tf1).

**Figure 1:**
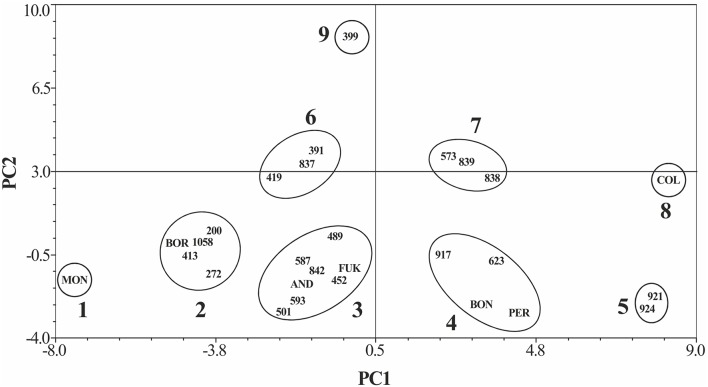
**Ordination the common bean genotypes studied according to the Principal Component Analysis (PCA)**. PC1, first principal component; PC2, second principal component.

Nine groups arose from the PCA ordination (Table 6, Figure 1). The x-axis (PC1) represents variation in emergence time and proportion of emergence in the open field. Genotypes located at the left side have earlier emergence and higher emergence proportion than those on the right side. The y-axis represents variation in the same variables in the controlled chamber, with genotypes at the lower side showing earlier emergence, and higher emergence proportion than those at the upper side. Montcau is the only genotype in group 1, five genotypes are included in group 2 (200 272, 413 1058, Borlotto), eight in group 3 (452, 489, 501, 587, 593, 842, Andecha, Fukuryu), four in group 4 (623, 917, Bonafema, Peregrina), two in group 5 (921, 924), three in group 6 (391, 419, 837), three in group 7 (573, 838, 839), only Collacia in group 8 and 399 in group 9.

**Table 6:** **Characteristics of the groups arising from the PCA of the common bean genotypes studied under different environments**.

**Group**	**Genotype**	**Genetic pool^a^**	**Seed color^b^**	**Seed size^c^**	**Emergence score^d^**
1	Montcau	M	w	m	g
2	200	A	w	m	g
	272	A	b	m	g
	413	A	b	x	g
	1058	M	w	l	g
	Borlotto	A	c	l	g
3	452	A	w	x	g
	489	A	c	s	g
	501	A	c	m	g
	587	M	w	m	g
	593	M	w	m	g
	842	A	w	x	g
	Andecha	A	w	x	g
	Fukuryu	A	c	l	g
4	623	M	w	m	m
	917	A	w	x	m
	Bonafema	A	w	x	m
	Peregrina	M	w	l	m
5	921	A	w	x	b
	924	A	w	x	b
6	391	A	c	l	m
	419	M	w	l	m
	837	A	c	l	m
7	573	M	c	m	m
	838	A	w	x	m
	839	A	w	x	m
8	Collacia	A	w	x	b
9	399	M	w	x	b

a *A, Andean; M, Mesoamerican*.

b *w, white; b, bicoloured; c, colored*.

c *x, extra-large (> 65 g 100 seeds^−1^); l, large (> 50 g 100 seeds^−1^); m, medium (> 35 g 100 seeds^−1^); s, small (< 35 g 100 seeds^−1^)*.

d *According to the PCA ordination in Figure 1: g, good; m, mediocre; b, bad*.

## Discussion

In controlled environments and field conditions, studies have shown that the rate of germination and seedling emergence linearly increases with temperature in several crop species including legumes, such as cowpea, soybean, chickpea, and peanut (Covell et al., 1986; Ellis et al., 1986; Mohamed et al., 1988; Craufurd et al., 1996; Awal and Ikeda, 2002). In this work we found that the variation in emergence in a range of temperatures was greater in controlled chamber than in open field. It can be argued that environmental conditions in the chamber are strictly controlled and the weighted average of temperature and RH had a range of variation wider than in the field. However, seeds in the field are exposed to the natural not controlled environmental variation that could explain the fact that the proportion of emergence is approximately half of the value in the controlled chamber. In field experiments, several factors can affect seed germination, and therefore the proportion of seedling emergence. One factor may be the presence of soil-borne pathogens (Burke and Kraft, 1974) that affect seeds when the emergence is delayed and the seed remains more time on the soil. Days to emergence in the field varied slightly with increasing temperature in later sowings, but this variation could be due also to other environmental factors such as soil properties and hydrological conditions.

Clear genotypes × temperatures interactions were found for emergence proportion and time to emergence. For instance, genotypes 573 and 623 showed contrasting emergence proportions in response to tf2 and tf3. Furthermore, time to emergence in the field was advanced only by 1 day in 399 compared to 6 days in Collacia.

The bean germplasm from the Iberian Peninsula is characterized by large and extra-large seeds with this trait displaying a broad sense heritability of 0.70 (Escribano et al., 1994; Casquero et al., 2006; González et al., 2006). In our study the seed size varies in average from 30 to 87 g 100 seeds^−1^ being 25% of the studied genotypes large and 43% extra-large seeded. The large seeds were the result of selection by farmers according to market and consumer preferences. Farmers use to germinate the large seeds in nurseries to ensure the process, then to transplant the seedlings to the open field. This practice could have favored selection for earlier emergence, but not for emergence proportion in the field. In fact, the PCA indicates a trend to lower emergence in large and extra-large seeded genotypes: the groups with good emergence (groups 1, 2, and 3) have seven large and extra-large seeded genotypes (50%) and seven medium and small seeded ones (50%). However, the groups with mediocre or bad emergence (groups 4, 5, 6, 7, 8, and 9) include 11 large and extra-large seeded genotypes (79%) and only three medium and small ones (21%).

With regard to market class and seed color, one of the factors that have been suggested as responsible for poor legume emergency in the field is imbibition damage caused by fast water uptake, resulting in cell death, solute leakage, and reduced emergence and growth. However, colored seeds which imbibed more slowly, suffer less damage than the white ones (Powell et al., 1986). Therefore, white seeded market classes are typically more susceptible to this damage. According to the PCA, the groups with good emergence (groups 1, 2, and 3) have eight white seeded genotypes, and six bicolor or colored, while the groups with bad emergence (4, 5, 6, 7, 8, and 9) included 11 white seeded genotypes and only three colored ones. These trends suggest that seed testa color could have some influence in seed germination and seedling emergence, in accordance with the results by other authors (Dickson, 1971; Powell et al., 1986).

Open field trials are the most reliable measure of low temperature tolerance since it is measured in the actual growth environment of the crop. In our research consistent comparable results were not observed between controlled chamber and field environments, in agreement with Kolasinska et al. (2000) and Khajeh-Hosseini and Rezazadeh (2011).

There is evidence that chilling tolerance at juvenile stages of development (germination, emergence, seedling growth) is under independent genetic control from chilling tolerance during reproductive development (Kemp, 1973; Dickson and Petzoldt, 1987; Melo et al., 1997). Coincidence of tolerance to high and low temperature stress has been reported in snap beans (Dickson and Boettger, 1984a,b), oat (Mashiringi and Harahwa, 1985), and maize (Yacoob and Filion, 1986). Porch (2001) found that snap bean lines developed for cold tolerance had significant positive general combining ability (GCA) under high temperatures for traits related to high temperature tolerance.

It is generally accepted that germination rate is affected by seed shape or size and, therefore, the quantity of nutrients stored in the seed (Cui et al., 2002; Hanley et al., 2003; Nonogaki, 2006; Kaya et al., 2008). In the experiments here reported, large seeded genotypes needed more days to seedling emergence than small seeded ones, both in the growth chamber and in the open field experiments, and showed lower emergence in the field under real growing conditions. This fact may be related to the history of the bean crop in southwest Europe, since its introduction in the early Sixteenth Century. Farmers probably selected large seeded common bean genotypes that resembled the Old World faba bean (*Vicia faba* L.) that was cultivated at that time, thus introducing the new crop as a novelty, which explains the name “faba” or “haba” used often in Spain for the common bean. In spite of the pleiotropic effect that seed size probably had in delaying germination and reducing emergency, famers continued to select large seeded genotypes of common bean such as the currently grown in the northwest of the Iberian Peninsula (Escribano et al., 1994; Rodiño et al., 2001, 2009).

Nine groups arose from the PCA representing variation in emergence time and proportion of emergence in the controlled chamber and in the open field. PCA indicates a trend to lower emergence in large and extra-large seeded genotypes. The genotypes 399 and 419 (large great northern market class), 921, 924, and Collacia (favada market class) are examples of white large seeded genotypes with poor germination in the field. In particular, according to the PCA, the favada market class genotypes (two in group 3 and six in groups 4, 5, 7, and 8), with very high market value, have lower proportion of emergence, probably due to the fact that farmers practice was to germinate the seed in the nursery, and afterwards transplant the seedling into the open field. Since then neither natural selection nor breeding had taken place to improve the germination and emergence of the favada market class genotypes or the large great northern ones whose seeds have similar market and uses by consumers. Nevertheless, in the present study some of these genotypes (e.g., 399, 419, 917, 921, and 924) showed high emergence (>80%) when germinated at low temperature in a controlled environment chamber. This was probably due to the fact that these genotypes were selected in the past to be germinated in stable environments, not in unstable open field conditions.

In the experiments here reported, the emergence process was assessed by time to emergence and the proportion of seeds producing emerged seedlings, with relevance in the conservation of germplasm in gene banks. In order to conserve the genetic structure of the original accessions, regeneration of germplasm in the field should be performed only when the results of the germination tests display low germination. Emergence in controlled conditions in climatic chamber resembles the viability test used in gene banks, whereas sowing in the field is used to regenerate accessions. According to our results the viability tests should be complemented with vigor tests that can provide better estimation of field emergence (Hampton and Tekrony, 1995). For the same reason, in regeneration processes of bean germplasm it would be advisable to germinate seeds under favorable conditions (e.g., in nurseries) before transplanting the seedlings into the field, in order to prevent (or minimize) genetic erosion due to the mortality of viable seeds.

According to our results, temperature conditions had a relevant role in the crop yield, together with the environmental factors. The plant productivity, which is the major expression of the genotypes fitness, hade the higher values when sowing and growing the plants under low temperature, decreasing when the temperature was increased. It is relevant also to link the yield with the process of emergence of seedlings at different temperatures in the open field and in the controlled chamber. Taken into account these results, the genotypes 272, 501, 593, and 1058, and the cultivar Borlotto had assembled the best conditions for early sowing achieving good yield performance.

In conclusion, seed germination, seedling emergence, plant growth, and crop yield under different temperatures are of relevance for the selection of common bean genotypes with better performance under stress temperatures, but also may lead to potential genetic erosion in germplasm collections. As a result of the screen of seedling emergence and phenotypic response of bean germplasm under a range of temperatures in controlled chamber and field conditions, some genotypes such as landraces 272, 501, 593, and the cultivar Borlotto were identified as temperature stress-tolerant at sowing time and seedling emergence with good agronomic performance and yield potential and they could be a valuable genetic material for breeding programs. Additionally, the efficiency of bean genebanks standard germination tests for predicting the performance of the seeds in the field was assessed comparing the emergence of bean seedlings under controlled environment and in open field. Regarding bean commercial traits, under low temperature at sowing time bean seeds reached larger size, and the crop yield was higher compared to warmer temperatures at this stage. Therefore, early sowing of bean is strongly recommended.

## Author contributions

AD: conception and experimental design of the work, including chamber, and field experiments; revising the work and approval of the version to be published. AR: experimental design of the work; acquisition and analysis of chamber and field data for the work; drafting and revising the work and approval of the version to be published. MS: interpretation of field and chamber data for the work; drafting and revising the work and approval of the version to be published. AG: statistical analysis of field and chamber experimental data; revising the work and approval of the version to be published. ML: interpretation of soil data for the work; revising the work and approval of the version to be published. IM: interpretation of germplasm data for the work; revising the work and approval of the version to be published. JK: analysis and interpretation of physiological data for the work; revising the work and approval of the version to be published.

## Funding

Research was supported by the projects AGL2014-51809-R and RFP2013-00001 from the Spanish Government (Ministerio de Economía y Competitividad) and AGI/CSIC I+D+I 2014 OTR00114 from the Galician Government-CSIC (Spain).

### Conflict of interest statement

The authors declare that the research was conducted in the absence of any commercial or financial relationships that could be construed as a potential conflict of interest.
