# Supplementary material for: Seedling Emergence and Phenotypic Response of Common Bean Germplasm to Different Temperatures under Controlled Conditions and in Open Field
Source: Front Plant Sci. 2016 Aug 2;7:1087. doi: 10.3389/fpls.2016.01087 (PMC4969293; doi:10.3389/fpls.2016.01087)
Supplement: Supplementary file 1 [file Table1.DOCX]

Table S1. Average and standard deviation (in parenthesis) of the temperature and relative humidity (RH) of the open field trials for evaluation of the temperature response of the common bean genotypes studied at different growth stages.

| Trial | Temperature (ºC)^a^ | | | | | | | | | | |  | RH (%)^a^ | | |
| --- | --- | --- | --- | --- | --- | --- | --- | --- | --- | --- | --- | --- | --- | --- | --- |
|  | Emergence | | |  | First trifoliate leaf | | |  | First flower | | |  | Emergence | First trifoliate leaf | First flower |
|  | M | Max | Min |  | M | Max | Min |  | M | Max | Min |  | M | M | M |
| tf1 | 14.2 (2.31) | 20.2 (4.03) | 8.7 (1.97) |  | 14.3 (2.19) | 20.4 (3.82) | 8.6 (1.87) |  | 15.6 (2.14) | 20.7 (3.47) | 10.7 (2.60) |  | 74.1  (7.63) | 73.5 (7.45) | 73.4 (7.36) |
| tf2 | 15.4 (1.97) | 20.3 (3.77) | 10.5 (2.51) |  | 16.0 (2.26) | 21.2 (4.03) | 10.9 (2.42) |  | 16.7 (2.04) | 21.5 (3.45) | 11.8 (2.37) |  | 71.2  (6.27) | 70.4 (6.48) | 72.9 (7.80) |
| tf3 | 16.6 (0.92) | 20.2 (1.54) | 13.1 (1.25) |  | 16.4 (0.82) | 20.6 (1.55) | 12.2 (1.83) |  | 17.9 (2.05) | 22.9 (3.53) | 12.6 (2.12) |  | 78.4  (4.63) | 76.3 (5.53) | 72.7 (8.90) |

^a^M=average, Max=maximum and Min=minimun
