# Supplementary material for: Seedling Emergence and Phenotypic Response of Common Bean Germplasm to Different Temperatures under Controlled Conditions and in Open Field
Source: Front Plant Sci. 2016 Aug 2;7:1087. doi: 10.3389/fpls.2016.01087 (PMC4969293; doi:10.3389/fpls.2016.01087)
Supplement: Supplementary file 2 [file Table2.DOCX]

Table S2. Mean value and standard deviation (SD) for some chemical properties of the soil of the experimental plot where the common bean genotypes studied were tested.

|  | **OM**^a^ |  | **pH** | |  | **P**^b^ |  | **K** | **Ca** | **Mg** | **Al** | **ECEC**^c^ | |
| --- | --- | --- | --- | --- | --- | --- | --- | --- | --- | --- | --- | --- | --- |
|  | **%** |  | **H_2_O** | **KCl** |  | **ppm** |  | **cmol(+)/kg** | | | | | |
| Mean | 4.2 |  | 5.7 | 4.6 |  | 139.0 |  | 0.6 | 3.7 | 0.4 | 0.7 | | 5.4 |
| SD | 0.30 |  | 0.18 | 0.18 |  | 11.21 |  | 0.07 | 0.57 | 0.07 | 0.22 | | 0.45 |

^a^OM=Organic matter by weight loss-on ignition

^b^P=Phosphorus availability by Olsen method

^c^ECEC=Efective cation exchange capacity
