# Supplementary material for: Seedling Emergence and Phenotypic Response of Common Bean Germplasm to Different Temperatures under Controlled Conditions and in Open Field
Source: Front Plant Sci. 2016 Aug 2;7:1087. doi: 10.3389/fpls.2016.01087 (PMC4969293; doi:10.3389/fpls.2016.01087)
Supplement: Supplementary file 3 [file Table3.DOCX]

Table S3. Mean, maximum and minimum values for time to emergence and proportion of emergence in the common bean genotypes studied under different environments.

|  | Emergence time (d) | | | Emergence proportion (%) | | |
| --- | --- | --- | --- | --- | --- | --- |
|  | t1 | t2 | t3 | t1 | t2 | t3 |
|  | Chamber | | | | | |
| Mean | 27.2 | 7.8 | 4.6 | 72.9 | 88.2 | 94.4 |
| Max | 31.3 | 11.0 | 9.3 | 96.7 | 100.0 | 100.0 |
| Min | 23.0 | 7.0 | 3.7 | 30.0 | 66.7 | 76.7 |
|  | Field | | | | | |
| Mean | 15.4 | 11.3 | 12.8 | 45.1 | 46.0 | 40.5 |
| Max | 20.5 | 23.5 | 14.0 | 75.0 | 58.3 | 50.0 |
| Min | 10.0 | 8.0 | 6.0 | 16.7 | 20.0 | 11.7 |
|  | Overall | | | | | |
| Mean | 21.3 | 9.5 | 8.7 | 59.0 | 67.1 | 67.4 |
| Max | 31.3 | 23.5 | 14.0 | 96.7 | 100.0 | 100.0 |
| Min | 10.0 | 7.0 | 3.7 | 16.7 | 20.0 | 11.7 |
